# In a Hospital

**Published:** 1889-08-03

**Authors:** 


### In a Hospital.

*"Miserere mihi Domine et exaudi orationem meum."*

Look in pity on Thy children,
Sick and sorrowful are we;
Thou who once didst heal the leper,
Miserere Domine.

Sad our lives, and often dreary,
Sickness on all sides we see;
Jesus, who didst heal the leper,
Miserere Domine.

Thou alone canst heal our weakness,
All our pains are known to Thee;
Jesus, who didst heal the leper,
Miserere Domine.

Teach us how to bear our burdens,
Till Thy mercy sets us free;
Jesus, who didst heal the leper,
Miserere Domine.

May Thy mercy cure us wholly,
But if, Lord, that may not be,
Still in death, as in our sickness,
Miserere Domine.

